# Managing Non-islet Cell Tumor Hypoglycemia in Hepatocellular Carcinoma With Radiation Therapy

**DOI:** 10.7759/cureus.76372

**Published:** 2024-12-25

**Authors:** Anthony J Chillo, Sylvester Homsy, Aye M Thida, Edwin Chiu

**Affiliations:** 1 Hematology and Oncology, State University of New York Downstate Health Sciences University, Brooklyn, USA; 2 Hematology and Oncology, Stanford University School of Medicine, Stanford, USA; 3 Medicine, State University of New York Downstate Health Sciences University, Brooklyn, USA

**Keywords:** case report, hepatocellular carcinoma, non-islet cell tumor hypoglycemia, paraneoplastic syndrome, radiation therapy

## Abstract

Non-islet cell tumor hypoglycemia (NICTH) is a paraneoplastic syndrome associated with non-mesenchymal-derived and epithelial tumors. A 37-year-old male with stage IVB hepatocellular carcinoma (HCC) and pulmonary metastases presented with recurrent hypoglycemia despite glucose supplementation. Laboratory findings revealed low insulin growth factor 1 (IGF-1) (15 ng/mL), elevated insulin growth factor 2 (IGF-2) (395 ng/ml), and an IGF-2:IGF-1 ratio of 26:1, consistent with NICTH. After ruling out other causes of hypoglycemia, including endocrine deficiencies and medication-induced hypoglycemia, the patient was managed with steroids and intravenous (IV) glucose. Due to the metastatic nature of the cancer, he was treated with atezolizumab and bevacizumab. Palliative radiation therapy (RT) was initiated to improve glycemic control. Following RT, hypoglycemic episodes decreased, allowing discharge with oral steroids. NICTH management remains challenging due to limited therapeutic options and variable treatment responses. Surgical resection is the standard treatment for NICTH; however, conservative approaches include steroid use, glucose supplementation, and recombinant growth hormone (GH). In this case, radiation was chosen to target the tumor and alleviate hypoglycemia, resulting in improved glycemic stability post-treatment. NICTH associated with HCC is a rare and challenging complication with significant morbidity. Early use of RT alongside systemic treatment may offer a viable strategy for managing NICTH and improving patient outcomes.

## Introduction

Non-islet cell tumor hypoglycemia (NICTH) is defined as hypoglycemia caused by a non-beta islet cell tumor and is a rare but serious complication seen in patients with mesenchymal-derived tumors and epithelial tumors [1]. According to The Endocrine Society Guidelines, investigation of NICTH should be evaluated in patients who meet Whipple’s triad (low plasma glucose levels, signs and symptoms of hypoglycemia, and relief of symptoms after increased plasma glucose levels) and when other causes such as medication-induced hypoglycemia, critical illness, organ failure, hormone deficiency, and endogenous hyperinsulinism hypoglycemia are ruled out [2]. Furthermore, NICTH is characterized by low levels of insulin, proinsulin, and beta-hydroxybutyrate. A diagnosis of NICTH is confirmed with the molecular ratio of insulin growth factor 2 (IGF-2)/insulin growth factor 1 (IGF-1) of 10. This case report describes a patient with metastatic hepatocellular carcinoma (HCC) admitted for hypoglycemia who was found to have NICTH and the use of radiation therapy (RT) to manage this paraneoplastic syndrome.

## Case presentation

**Figure 1:**
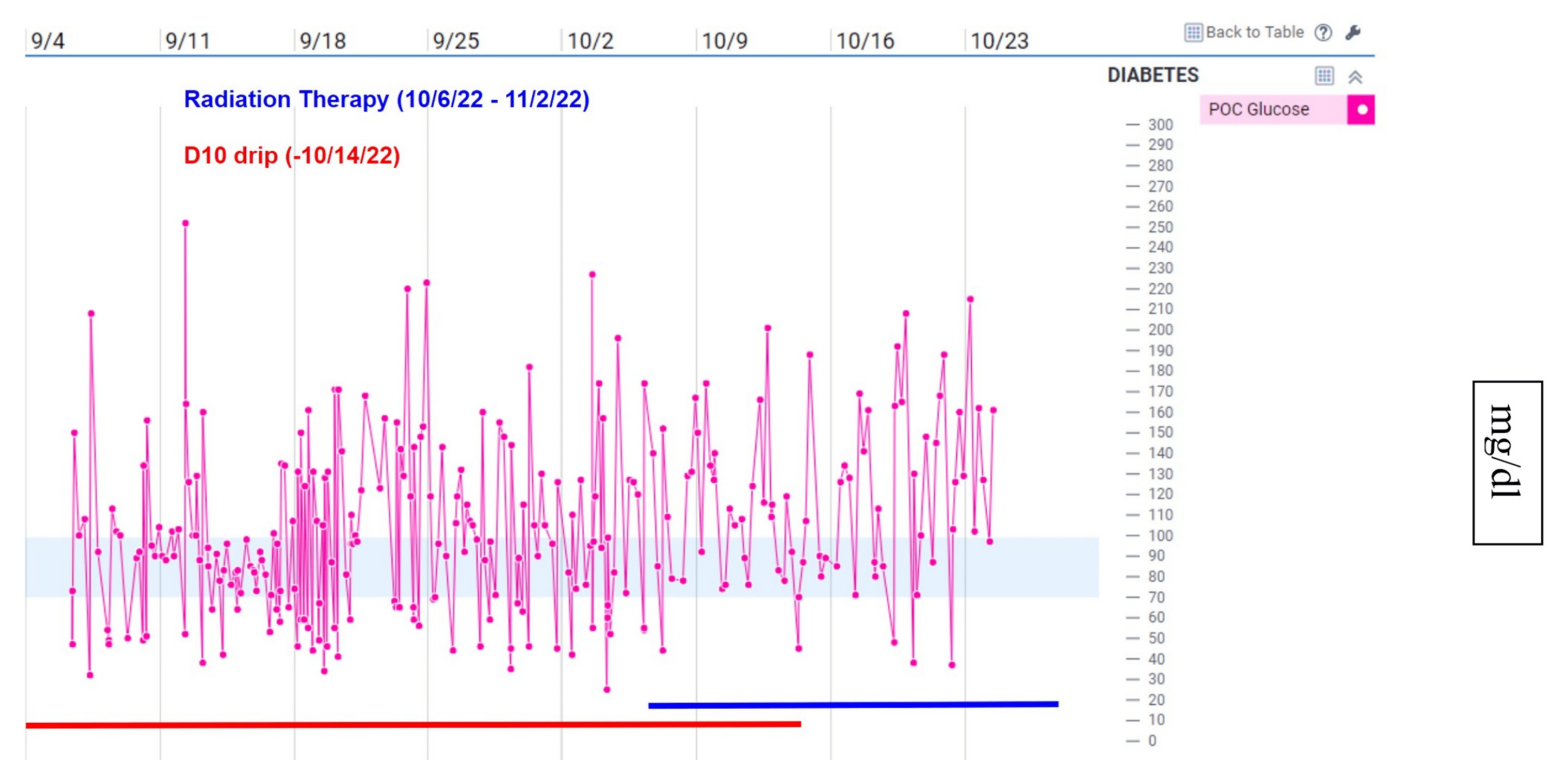
Fingerstick glucose measurements with D10 drip and radiation therapy timeline

**Figure 2:**
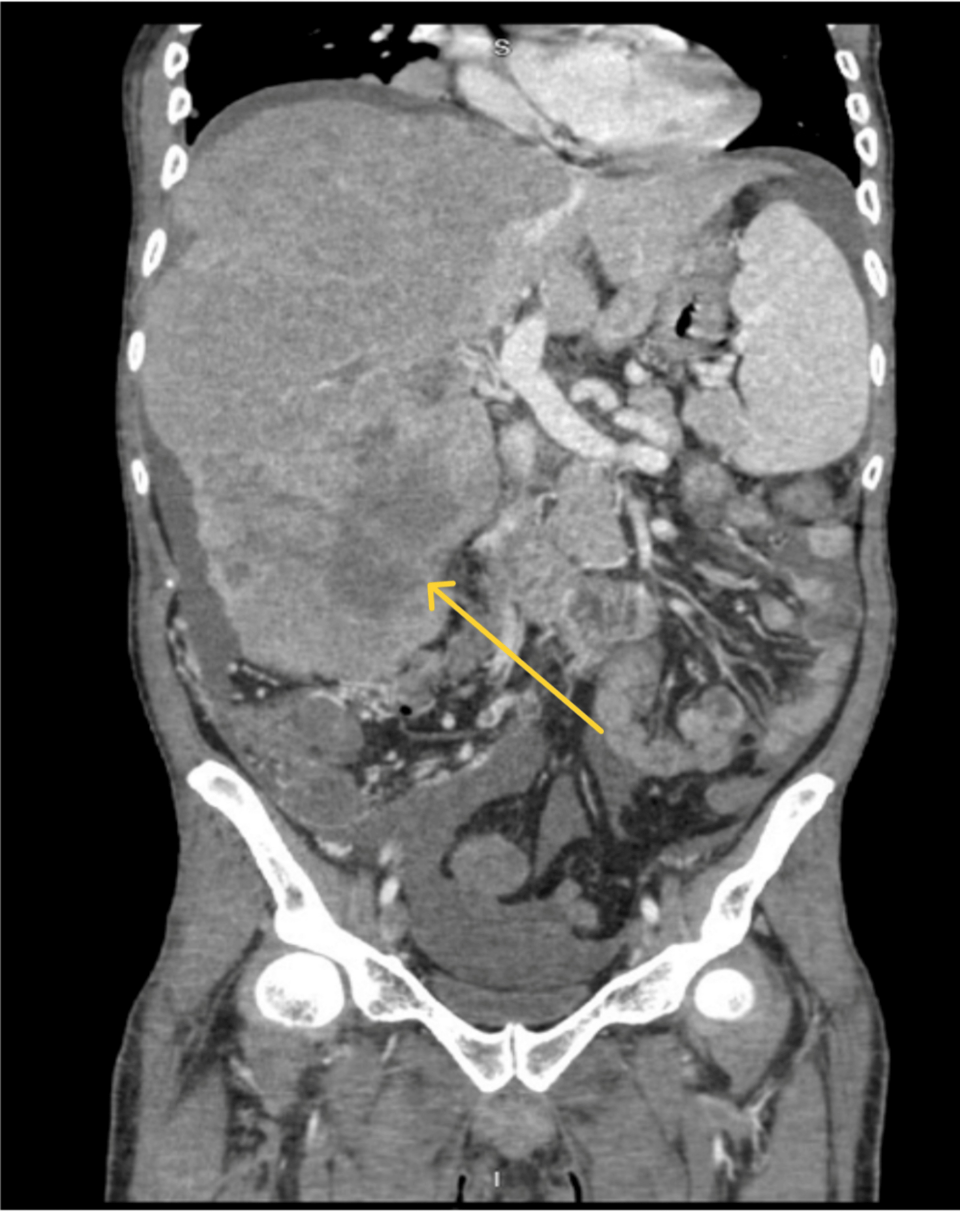
Computed tomography (CT) scan of the abdomen, coronal view showing enlarged liver with liver lesions (yellow arrow)

**Figure 3:**
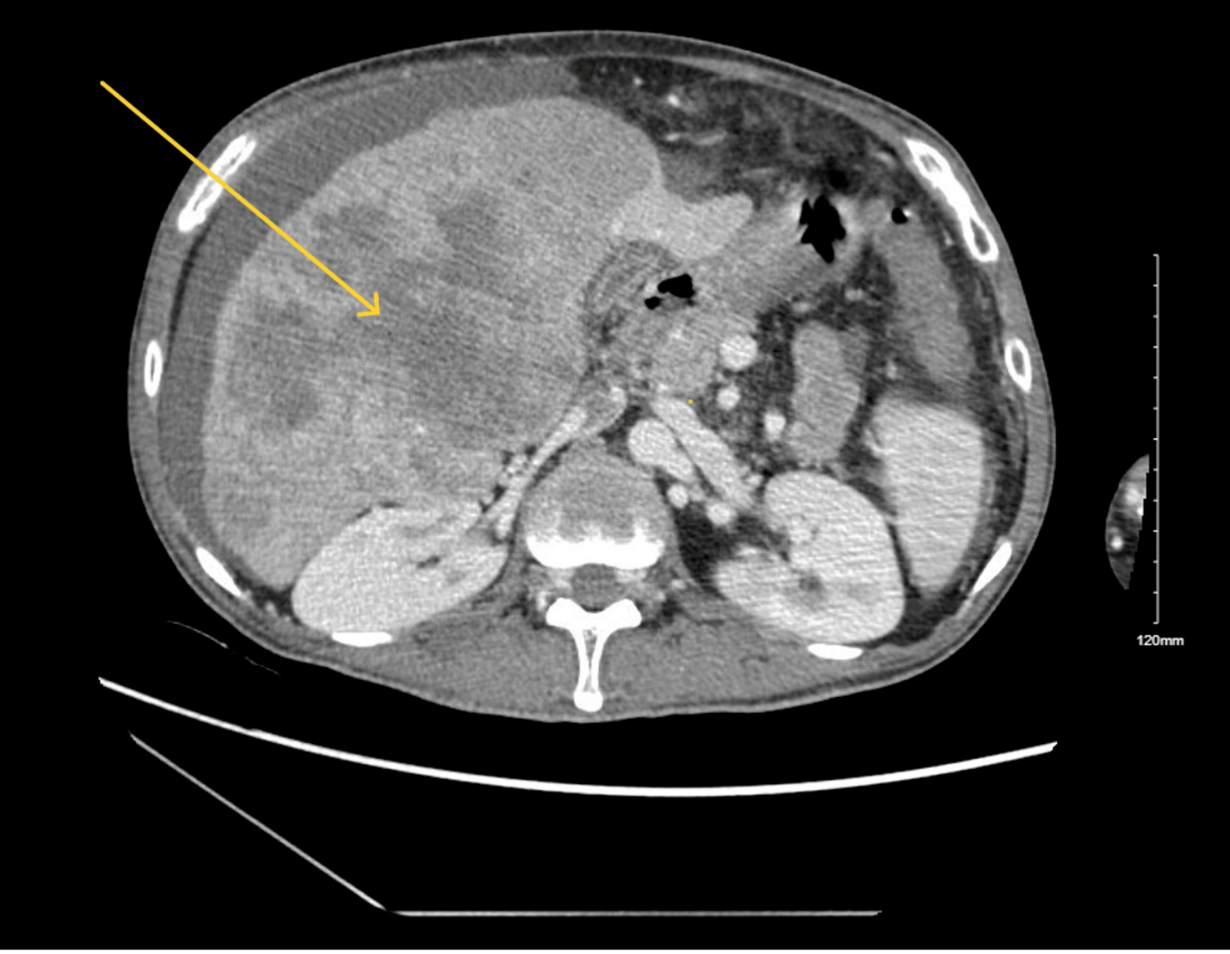
Computed tomography (CT) scan of the abdomen, transverse view showing enlarged liver with liver lesions (yellow arrow)

A 37-year-old male patient from Trinidad was found at home with altered mental status and was transported to the hospital by ambulance. The patient's medical history included Hepatitis B cirrhosis, trace varices, and stage IVB (cT4N0M1) HCC with pulmonary metastases (Figures 2-3). The patient's HCC was staged using the American Joint Committee on Cancer Tumor Nodes Metastasis system. The patient had low blood sugar (glucose level of 35 mg/dl) upon arrival, which improved with intravenous (IV) dextrose. The patient continued to experience hypoglycemia (47 mg/dl) in the emergency department and was admitted for further management. The patient's laboratory results revealed low levels of IGF-1 (15 ng/ml), normal levels of IGF-2 (395 ng/ml), and an elevated IGF-2-to-IGF-1 ratio of 26:1, indicating non-islet cell tumor hypoglycemia. Thyroid dysfunction, adrenal insufficiency, and the use of hypoglycemic agents were effectively ruled out by laboratory investigations. The patient was treated with steroids and IV glucose to manage his hypoglycemia and continued outpatient systemic therapy with atezolizumab and bevacizumab. Due to the metastatic nature of the patient's malignancy, resection was not an option, and palliative RT was recommended to reduce tumor burden and improve blood glucose control. After starting RT, the frequency of hypoglycemic episodes reduced, and he finally maintained euglycemia without IV glucose (Figure 1). He completed 14 of 15 sessions of RT in the hospital, and he could be discharged home with oral steroids.

## Discussion

As the incidence of tumors associated with NICTH rises, there is a growing demand for an enhanced treatment protocol to address this condition. In order to improve treatment, the underlying pathophysiology of this condition must be understood. The initial hypothesized mechanism of NICTH was glucose utilization by large tumors [3]. The most accurate hypothesis is that NITCH is caused by tumor overexpression of IGF-2, both mature IGF-2 and incompletely processed IGF-2, which has potent insulin-like activity causing hypoglycemia [4,5]. NICTH management is challenging because of the limited therapeutic options available, unpredictable responses to treatment, and adverse side effects of the treatment. The proposed definitive treatment of NICTH is complete tumor resection [6-10]. In this case and many other cases, patients are not considered a good candidate for surgical resection in which medical treatment options must be examined. The medical treatments for NICTH are oral prednisolone, and intermittent use of glucose supplements can be used to maintain glucose homeostasis [1,7]. Other treatment options include treatment with human growth hormone which increases the mean glucose levels and a small increase in insulin-like growth factors. Furthermore, it is proposed that low-dose prednisone and recombinant human GH can be used as a long-term therapy for hypoglycemia [11]. Though these treatments treat hypoglycemia, they do not treat the underlying cause and therefore are not a definitive treatment. One alternate treatment option that showed promise was intrahepatic adriamycin. This treatment restored elevated levels of E-21 to normal and increased IGF-1 levels, while IGF-2 levels remained increased [12]. Radiation was considered because the radiation can be used to treat the solid tumor and radiation has shown success in treating NICTH caused by other tumors [13]. This treatment option was chosen because it would aid in the reduction of the tumor and treat the immediate glucose dysregulation. After starting RT, the number of hypoglycemic episodes was reduced, and he finally achieved a euglycemic state without IV glucose.

## Conclusions

NICTH is a rare paraneoplastic syndrome seen in hepatocellular carcinoma conferring a worse prognosis; its management remains challenging with the majority of the patients succumbing to the disease. RT could play a role if used early on and could be more effective in combination with other treatment modalities.
